# *Mosquito* (*MS*), a DD37E Family of *Tc1*/*Mariner*, Displaying a Distinct Evolution Profile from DD37E/*TRT* and DD37E/*L18*

**DOI:** 10.3390/genes14071379

**Published:** 2023-06-29

**Authors:** Kuilin Xiang, Mikhail Puzakov, Shasha Shi, Mohamed Diaby, Numan Ullah, Bo Gao, Chengyi Song

**Affiliations:** 1 College of Animal Science and Technology, Yangzhou University, Yangzhou 225009, China; 2 A.O. Kovalevsky Institute of Biology of the Southern Seas of RAS, Lenninsky Ave, 38, Moscow 119991, Russia

**Keywords:** transposon, *Tc1/Mariner*, DD37E/*Mosquito*, horizontal transfer

## Abstract

Diverse *Tc1/mariner* elements with the DD37E signature have been detected. However, their evolutionary relationship and profiles are largely unknown. Using bioinformatics methods, we defined the evolution profile of a *Tc1/Mariner* family, which harbors the catalytic domain with the DD37E signature, and renamed it DD37E/*Mosquito* (*MS*). *MS* transposons form a separate monophyletic clade in the phylogenetic tree, distinct from the other two groups of elements with the DD37E signature, DD37E/*L18* and DD37E/*TRT* (transposon related to *Tc1*), and represent a very different taxonomic distribution from that of DD37E/*TRT*. *MS* is only detected in invertebrate and is mostly present in Arthropoda, as well as in Cnidaria, Ctenophora, Mollusca, Nematoda, and Platyhelminthes, with a total length of about 1.3 kb, containing an open reading frame (ORF) encoding about 340 amino acids transposases, with a conserved DD37E catalytic domain. The terminal inverted repeat (TIR) lengths range from 19 bp to 203 bp, and the target site duplication (TSD) is TA. We also identified few occurrences of *MS* horizontal transfers (HT) across lineages of diptera. In this paper, the distribution characteristics, structural characteristics, phylogenetic evolution, and horizontal transfer of the *MS* family are fully analyzed, which is conducive to supplementing and improving the *Tc1/Mariner* superfamily and excavating active transposons.

## 1. Introduction

The vast majority of eukaryotes have a high proportion of repeated sequences in their genomes, which are mainly derived from transposable elements (TEs). Eukaryotic TEs are divided into retrotransposons (TE class I) and DNA transposons (TE class II). Accepted groups of Class I TEs are long terminal repeat (LTR) retrotransposons, including endogenous retroviruses, non-LTR retrotransposons, tyrosine recombinase retrotransposons, and *Penelope*-like elements. All of these TEs use reverse transcriptase for amplification but differ in the catalytic components responsible for integration into the host genome. DNA transposons are known to use three transposition mechanisms: DDD/E transposase, tyrosine recombinase, and HUH endonuclease in combination with helicase [1,2,3].

TEs encoding DDD/E transposase are the predominant group of DNA transposons and are classified in Repbase into 21 superfamilies (*Tc1/mariner*, *hAT*, *MuDR*, *EnSpm*/*CACTA*, *piggyBac*, *P*, *Merlin*, *Harbinger*, *Transib*, *Polinton*, *Kolobok*, *ISL2EU*, *Sola*, *Zator*, *Zisupton*, *Ginger1*, *Ginger2/TDD*, *Academ*, *Novosib*, *IS3EU,* and *Dada*) [4,5]. Additionally, as a result of recent studies, *pogo* elements (formerly included in *Tc1/mariner*) are now classified as a separate superfamily [6]. In addition, a new *Sailor* superfamily close to the *Tc1/mariner* elements was found [7]. Based on the similarity of catalytic domains and other highly conserved motifs, some superfamilies can be combined: *Harbinger* and *ISL2EU*; *MuDR*, *Rehavkus*, P, *hAT*, and *Kolobok*; and *EnSpm*, *Mirage*, *Chapaev*, and *Transib* [8].

The *Tc1/mariner* superfamily is one of the most widespread and diverse superfamilies of DNA transposons [9]. Phylogenetic analysis shows similarities between *Tc1/mariner* transposases and RNase H and *HIV-1* integrases [10]. The *Tc1/mariner* superfamily is related to the *IS630* bacterial family [11]. In this regard, they are also combined into the infraclass *IS630/Tc1/mariner* (*ITm*), which also includes the superfamilies *pogo*, *Sailor*, and *Gambol* [6,7].

The elements of the *Tc1/mariner* superfamily are divided into several families, many of which are well-studied: DD34-38E/*TLE* (*Tc1*-like elements), DD34D/*MLE* (*mariner*-like elements), DD37D/*maT*, DD41D/*Visitor* (*VS*), DD39D/*Guest* (*GT*), and DD37E/*L18* [9,12,13,14,15]. At the same time, there is a group of elements with the DD37E signature, which was identified quite a long time ago [12] but has not yet been practically studied. Two more groups of *Tc1*/mariner elements with the DD37E signature are also known. The DD37E/*TRT* (*Tc1*-like elements) subfamily is well-defined and shows a taxonomic distribution among fungi, protists, and animals [16]. The second group (named *L18*) forms a separate clade from DD37E/*TRT* and has been found in the genomes of some bivalves, crustaceans, insects, mites, fish, cnidarians, and echinoderms [17]. The structure, distribution, diversity, and evolution of DD37E/*L18* transposons have been studied in detail only in cnidarians [15]. Both of these groups obviously differ from elements of the DD37E clade, described by Shao and Tu [12], in mosquitoes and conventionally designated *mosquito* in some works [17,18] and *ITmD37E* in others [3]. In order to avoid further discrepancies, we have assigned the single name, *Mosquito* (*MS*), to this monophyletic clade.

In the current study, we systematically explored the evolutionary profiles of *Mosquito*, including taxonomic distribution, structural organization, and evolutionary dynamics. In addition, we also determined the phylogenetic relationships of three groups of DD37E transposons (*TRT*, *L18*, and *MS*), based on reference families of other *Tc1/mariner* members.

## 2. Materials and Methods

### 2.1. DD37E/MS Mining

To assess the taxonomic distribution of DD37E/*MS* transposons in genomes, the reference transposase sequences from the genome of Anopheles gambiae (336 aa, GenBank access number AAAB01008948.1:27012-28318) were used as a query to search against the whole-genome shotgun contig database (WGS), which includes all of the sequenced genomes of prokaryotes and eukaryotes, at the National Center for Biotechnology Information (NCBI) using TBlastN with an E-value of 1 × 10^−4^. The best hits were extracted with 2 kb flanking sequences and downloaded to define the boundaries of *MS*s, since most *ITm* families contain TIRs less than 2 kb, as previous studies reported, such as DD35E/*TR*, DD36E/*IC*, DD38E/*IT*, DD37D/*maT*, and DD39D/*GT*. Then, the boundary signs (TSDs and TIRs) of *MS* were manually determined, based on the alignment of *MS* copies. The consensus sequences of *MS* in each genome were reconstructed using multiple alignments of copies (≥5 copies) by using the online emboss explorer (http://www.bioinformatics.nl/emboss-explorer/, accessed on 16 March 2023). Otherwise, the best hit of *MS* transposase was used as the representative sequence of *MS* for the species in case the copy number was less than 5, or it was hard to derive the consensus sequence (too many truncated copies). The representative sequence, or the consensus sequence, of *MS* was searched against its host genome to estimate the copy number, which was calculated as the BlastN hits with 80% identity and 40% coverage of *MS*. The obtained transposases (≥200 aa) were used for multiple alignment and phylogenetic analyses to define the classification. The new sequences identified were then used as queries to recognize more *MS* elements. In addition, transposons with a very low copy number in the genome, which might be false-positive hits resulting from sequence contamination, were verified further by mapping the flanking sequences of the transposon insertion to the host genome or to the genomes of closely related species; the unmapped transposons were designated as sequence contamination and were excluded from the analysis.

### 2.2. DD37E/MS Sequence Analyses

Transposase coding sequences of DD37E/*MS* used in the present study were predicted by Genscan (http://hollywood.mit.edu/GENSCAN.html, accessed on 21 March 2023). Putative nuclear localization signal (NLS) motifs were predicted using PSORT II, as provided on the PSORT server (http://psort.nibb.ac.jp/, accessed on 23 March 2023), and the secondary structures and motifs of the transposases were predicted using the PSIPRED program (http://bioinf.cs.ucl.ac.uk/psipred/, accessed on 27 March 2023), Pfam (http://pfam.xfam.org/, accessed on 28 March 2023), and the HMMER web server. Multiple alignments of these elements were created by MAFFT v. 7.310. Shading and minor manual refinements of these aligned sequences were deduced using GeneDoc. The WebLogo 3 server (https://weblogo.threeplusone.com/create.cgi, accessed on 31 March 2023) was used to create the logo representation of the TSD and TIR sequences.

### 2.3. DD37E/MS Phylogenetic Analysis

The most conserved “DDE/D” domain of the identified *MS* (about 140 amino acids) transposases and reference *Tc1/mariner* families were aligned using the MAFFT program (v. 7.310). The phylogenetic trees were inferred with an ultrafast bootstrap value of 1000 using the maximum likelihood method within the IQ-TREE program. The best-suited amino acid substitution model for the data was the LG+I+G4 model, according to BIC, which was selected by Model Finder embedded in the IQ-TREE program. Furthermore, the Bayesian method was also applied to generate the phylogenetic tree, with the best-suited amino acid substitution model of LG+G+F, which was determined by BIC and selected by Protest (v. 3.4.2). Sequence identities between the *MS* family and six other families were measured by pairwise comparisons of DDE domains using the Bioedit software.

### 2.4. DD37E/MS HT Analyses

Putative HT events between organisms were detected based on pairwise distances and pairwise identities between the various organisms. Pairwise distances between the different animal species included in this study were calculated for the *MS* and the selected host gene sequences to test the HT hypothesis, which is well-established for DNA transposon HT events detection [7,19]. *MS* sequence identities greater than 70% or less than 70% were excluded from the HT analysis. Species that could not find the CDS region of the selected host gene sequences in the NCBI database were not included in this calculation. In this study, we used four host genes, which were RPL3 (60S ribosomal protein L3), RPL5 (60S ribosomal protein L5), Hsc70-4 (heat shock 70 kDa protein cognate 4), and Tub3 (tubulin β-3). When the genetic distance between ribosomal protein genes was greater than the genetic distance between *MS* transposons, and the sequence identity of *MS* between species was more than 70%, the horizontal transmission of *MS* between different species was designated. Multiple alignments of *MS* and the selected host gene sequences were conducted by using the MAFFT v. 7.310 program. Comparison distances between the host genes and transposons were calculated using MEGA software (v. 7.0.26), based on two aligned files (pairwise deletion and maximum composite likelihood). The identity calculation *MS* transposon was calculated using the Bioedit software. In addition, we constructed transposon and host phylogenetic trees to highlight phylogenetic incongruences that resulted from HT events. By comparing genetic distances and combining them with phylogenetic trees, we could infer the occurrences of horizontal transmissions.

## 3. Results and Discussion

### 3.1. Abundance DD37E/MS Transposons

**Figure 1:**
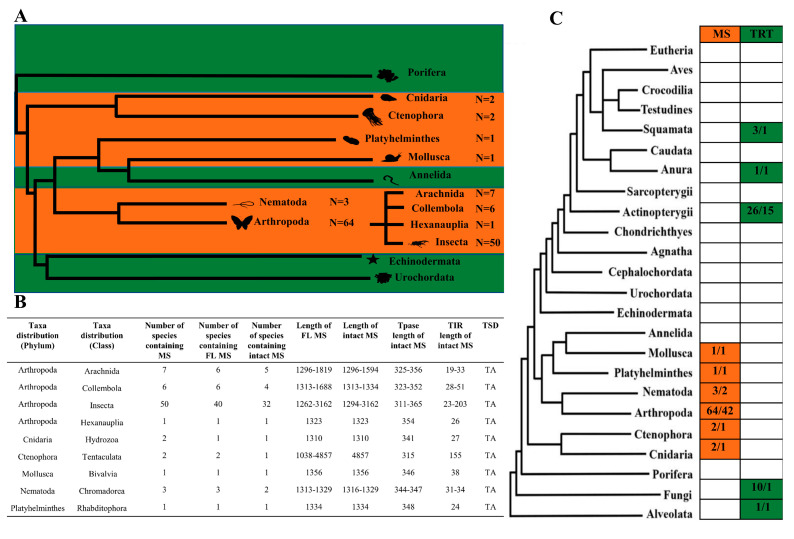
Taxonomic distribution of DD37E/*MS*. (**A**) Taxonomic distribution of DD37E/*MS* elements in the invertebrates. The numbers next to the animal silhouettes represent the number of *MS* elements detected in the species of each lineage. (**B**) Description of *MS* elements in 6 lineages, including the number of species with these elements, full-length (FL) elements, amino acid (aa) length of the transposase (Tpase), and lengths of terminal inverse repeats (TIRs). (**C**) Taxonomic distributions of *MS* and *TRT*. The number before the slash indicates the number of transposons, and the number after the slash indicates the number of intact transposons.

The study of the prevalence of *Mosquito* transposons among organisms showed that TEs were present in a number of large taxa (Figure 1). *MS* transposons were identified in ctenophores (Ctenophora), coelenterates (Cnidaria), flatworms (Platyhelminthes), mollusks (Mollusca), nematodes (Nematoda), and arthropods (Arthropoda). Among sponges (Porifera) and annelids (Annelida), as well as echinoderms (Echinodermata) and tunicates (Urochordata), *MS* elements were not found. Despite their wide presence on the taxonomic tree, *MS* showed a limited presence within taxonomic groups. Among the abundance of genomes available for study, *MS* was found only in some of them. Additionally, if we talk about full-sized and potentially functional *MS*, then this number was even lower. Such an evolutionary picture might indicate the ancient origin of *MS* transposons. In this case, *MS* was an ancient group whose ancestor entered the genomes of metazoans after the divergence of Eumetazoa and Porifera or even earlier. Further, it was mostly lost, leaving separate “traces of its presence” in large taxa. This scenario corresponds to the TE life cycle model, which involves a series of stages: invasion, proliferation, diversification, degradation, and elimination [20,21].

Another possibility is that the distribution on the taxonomic tree may be the result of multiple horizontal transfer (HT) events. HT is the process by which TEs can leave the host genome and become incorporated into the genome of another organism. In this case, invasions can be both intra- and interspecific. The HT phenomenon has been repeatedly described for transposons of the *Tc1/mariner* superfamily. In *Drosophila* genomes, the presence of *mariner* in *Zaprionus* may result from horizontal transfer. In ant genomes, the existence of horizontal transfer events has been reported for the *Tnigmar-Az mariner*. In the *R. prolixus* genome, at least 10 cases of horizontal transfers were found, supporting the idea that host/vector relationships played a pivotal role in the transmission and subsequent persistence of transposable elements [22,23,24]. In addition, it has been suggested that the level of activity of *mariner*-like transposons may also play an important role in their taxonomic distribution [25].

### 3.2. Phylogenetic Position of the DD37E/MS Family

**Figure 2:**
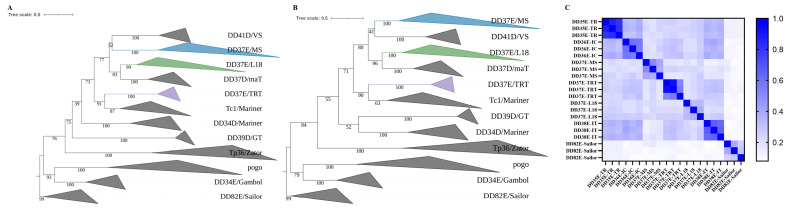
Phylogenetic position of the DD37E/*MS* superfamily. (**A**) This phylogenetic tree was generated based on DDE domains using the maximum likelihood method in the IQ-TREE program with an ultrafast bootstrap approach (1000 replicates). The reference families and elements included *Tc1*, DD34E/*Gambol*, DD37E/*TRT*, DD34D/*mariner*, DD37D/*maT*, DD39D/*GT*, DD41D/*VS*, DDxD *pogo*, DD37E/*L18*, *TP36*, and *Zator*. DD82E/*Sailor* was used as an outgroup. The uncollapsed tree is presented in Figure S1. (**B**) This phylogenetic tree was generated based on DDE domains using the Bayesian method in the MrBayes program. The uncollapsed tree is presented in Figure S2. (**C**) Sequence identities between the *MS* family and six other families. The sequence identities were measured by pairwise comparisons of DDE domains. Detailed data are listed in Table S2.

As a result of the phylogenetic analysis based on the IQ-TREE and MrBayes programs, which included *MS* elements and known representatives of other groups of the *IS630/Tc1/mariner* (*ITm*) infraclass, as well as elements *TP36*, *Zator*, and DD82E/*Sailor* (as outgroups), it was found that *MS* transposons were a separate monophyletic group (Figure 2A,B). At the same time, this family was not related to other groups of elements with the DD37E signature—DD37E/*L18* and DD37E/*TRT*. The dendrogram shows the phylogenetic proximity between *MS* and DD41D/*VS*, where DD37E/*L18* is closer to DD37D/*maT*, and DD37E/*TRT* is clearly part of the larger *Tc1* family.

A pairwise comparison of transposons also showed that *MS* sequences were most different from other representatives of the *ITm* infraclass (except *Sailor*) (Figure 2C). This also confirmed that the DD37E/*MS* elements belonged to a separate unique group with their own evolutionary history.

### 3.3. Structure of DD37E/MS Transposons

The *Tc1/mariner* superfamily is a large and diverse group and includes TEs with different characteristics [9,12,13,14,15]. Nevertheless, it is possible to single out the main feature characteristic of the representatives of this group.

The lengths of *Tc1/mariner* elements are, as a rule, from 1 to 3 kbp, but there are also longer variants. The *Tc1/mariner* transposons are flanked by terminal inverted repeats (TIRs), which can be up to 1900 bp in length but are usually in the range of 20–50 bp [26,27]. Some TEs also have so-called sub-terminal inverted repeats (SIRs). The lengths of the identified SIRs vary from 175 to 1403 bp [27]. *Tc1/mariner*, as a rule, contains a single intronless open reading frame (ORF) encoding the transposase enzyme, the length of which varies from 350 to 650 aa. Some representatives of *Tc1/mariner* (in particular *TLEWI*) have 3–5 introns [28]. The transposase of *Tc1/mariner* elements is characterized by the presence of a DNA-binding domain (PAIRED) and a catalytic domain (DDE/D). The PAIRED domain is located in the N-terminal part of the transposase and consists of six α-helices [29]. The DDE/D domain has endonuclease and ligation activities, allowing for the excision and insertion of TEs. It is located at the C-terminus of the transposase. Additionally, most transposases have detectable NLS (nuclear localization signal) motifs, which are supposed to ensure the transport of the enzyme from the cytoplasm to the nucleus [30,31].

**Figure 3:**
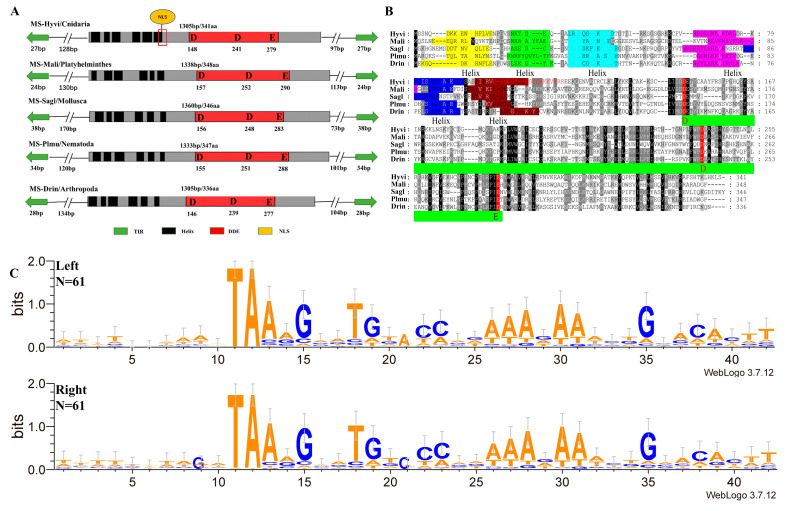
Schematic representation of the structure of DD37E/*MS* transposons. (**A**) Structural organization of *MS* elements. The green arrows represent TIR, the black rectangles represent HTH motifs, the red rectangles represent the catalytic domains, and the gray regions represent transposase (Tpase). (**B**) Alignment of the domains of *MS* transposases. We selected five representative species, that is, one Cnidaria, one Platyhelminthes, one Mollusca, one Nematoda, and one Arthropoda. For species abbreviations, MS-Hyvi: *Hydra viridissima*, MS-Mali: *Macrostomum lignano*, MS-Sagl: *Saccostrea glomerate*, MS-Plmu: *Plectus murrayi*, and MS-Drin: *Drosophila innubila*. (**C**) The WebLogo 3 server (https://weblogo.threeplusone.com/create.cgi, accessed on 15 April 2023) was used to create the logo representation for the left and right flanks (10 bp) and TA and TIR (30 bp) sequences (1–10 bp on behalf of the flanks, 11–12 bp on behalf of TSD, and 13–42 bp on behalf of the TIR). The value 2 (log2 4) on the y-axis stands for maximum possible frequency, and the N represents the number of sequences used for the logo generation.

An analysis of the structural features of *MS* showed that the predominant proportion of elements had lengths of 1300 to 1800 bp, with short TIRs (19–51 bp). At the same time, there were variants whose total length exceeded 4000 bp (Figure 1B). All elements that retained TIR had the classic [9] *Tc1/mariner* TSD transposon TA dinucleotide, and several motifs of TIRs were highly conserved (Figure 3C). The transposase encoded by ORF did not exceed 370 aa (Figure 1B). A total of 48 *MS* sequences contained complete transposases flanked by end TIRs, which were designated as the intact *MS*s, and all *MS* transposons had PAIRED and DDE/D domains (Figure 3A,B and Supplementary Table S3), while NLS was detected in 28 *MS*s. The position of the α-helices of the PAIRED domain in *MS* identified in representatives of distant taxa had some differences (Figure 3B) but generally corresponded to the characteristics of the *Tc1/mariner* superfamily. Meanwhile, when comparing the domain structures of transposases *MS*, *TRT*, and *L18* (Figure S3), visible differences in the positions of the third and sixth α-helices of the PAIRED domain were revealed. What effect this had on transposase functionality is still unknown.

### 3.4. HT Analysis of DD37E/MS Transposons

**Figure 4:**
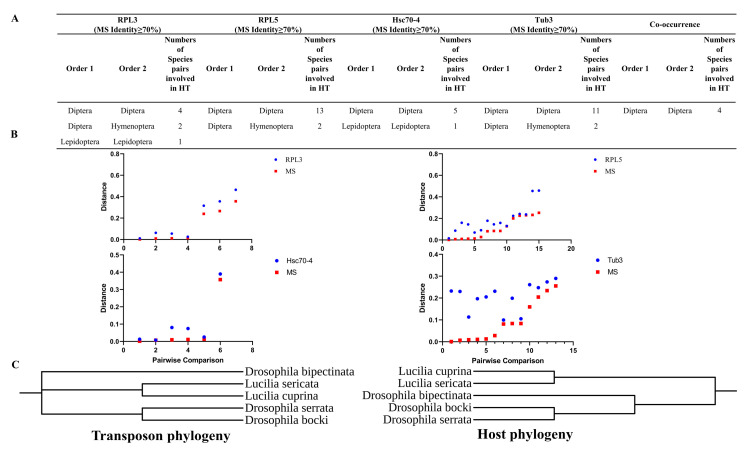
HT analysis of DD37E/*MS* transposons in invertebrates. (**A**) HT events of *MS* based on identity and genetic distance. (**B**) Graph illustrating the pairwise distances of *MS* and RPL3, RPL5, Hsc70-4, and Tub3 between the species included in this study. The distances were obtained from all possible pairwise comparisons. Detailed data are listed in Table S2. (**C**) Transposon and host phylogenetic tree. The host phylogenetic tree was created by using Time tree (http://timetree.org, accessed on 20 April 2023).

To identify possible events of horizontal transfer of *MS* transposons, we used a pairwise comparison of genetic distances between host genes and transposons, which has been used to identify the HT events of DNA transposons [7,13,32]. For comparison, RPL3, RPL5, Hsc70-4, and Tub3 were selected, which were most suitable for assessing the genetic distance between species as host genes. These genes showed higher sequence identities and greater lengths, as well as wider taxonomic distributions of a single genomic copy [7]. Only the species pairs sharing high sequence identifies (>70%) of *MS* transposons experienced HT events and remained for further analysis. Then, HT was detected and designated by cases where the genetic distances of *MS* transposons were lower than those of host genes between species, which are summarized in Figure 4A,B. Finally, the HT events between these species were also supported by transposon and host phylogenetic analysis (Figure 4C and Supplementary Table S4).

In bacteria and archaea, HT is a widely recognized mechanism for the exchange of genetic material [33], while HT is less common in eukaryotes [34]. HT transposons play a large role in their widespread distribution and evolution. However, the “behavior” (life cycle) of DNA transposons, which includes uneven rates of evolution, degeneration, and elimination, creates certain difficulties in detecting HT events [35,36]. Transposable elements that possess mobility and the ability to colonize and proliferate in new hosts contribute to widespread HT events in eukaryotes [36]. Although quite a lot of cases of HT have been described in elements of the *Tc1/mariner* superfamily [22,23,24], here, we found that very few species are involved in the HT events of *MS* transposons, and HT events were recorded in elements of *MS* for the first time.

## 4. Conclusions

The evolution profile of DD37E/*Mosquito*, which is a family of *Tc1/Mariner* transposons, was well-defined in this study. It displayed a distinct structural organization and phylogenetic position, compared with the known groups of this superfamily, were only distributed in invertebrates, and displayed a low frequency of HT events. When summarizing the data obtained as a result of HT analysis, data on the distribution of *MS* among eukaryotes, as well as the number of copies in genomes, we tend to assume that *MS* transposons are an ancient group whose ancestor entered the Metazoa a very long time ago. Associated with this is the single presence of *MS* in large taxa such as Ctenophora, Cnidaria, Platyhelminthes, Mollusca, and Nematoda. This observation not only improves our understanding of the evolution of the *Mosquito* superfamily, it also expands our understanding of the diversity of *ITm* transposons and updates the classification of this group.

## Data Availability

All data needed to evaluate the conclusions in this paper are present, either in the main text or the Supplementary Materials and are available on request from the corresponding author.

## Supplementary Materials

The following supporting information can be downloaded at: https://www.mdpi.com/article/10.3390/genes14071379/s1, Figure S1: Uncollapsed phylogenetic position of the DD37E/*MS* family (maximum likelihood method, IQ-TREE). Figure S2: Uncollapsed phylogenetic position of the DD37E/*MS* family (Bayesian method, MrBayes). Figure S3: Alignment of the domains of DD37E/*L18*, DD37E/*TRT*, and DD37E/*MS* transposases. The red font represents NLS. Table S1: Details of DD37E/*MS*, including species distribution, protein length, TIR, TSD, etc. Table S2: Details of sequence identities between the *MS* family and six other families. Table S3: PAIRED, DDE/D domains, and NLS of intact *MS*s. Table S4: HT event statistics of *MS*, including the pairwise genetic distance of the *MS* transposon, the pairwise genetic distance of the host gene (RPL3, RPL5, Hsc70-4 and Tub3), the pairwise identity of the transposon, the ratio of the genetic distance of the host gene to the genetic distance of the *MS*, and the order of the species involved. Table S5: Accession numbers of the host gene.
