# Supplementary material for: Mosquito (MS), a DD37E Family of Tc1/Mariner, Displaying a Distinct Evolution Profile from DD37E/TRT and DD37E/L18
Source: Genes (Basel). 2023 Jun 29;14(7):1379. doi: 10.3390/genes14071379 (PMC10379824; doi:10.3390/genes14071379)
Supplement: Supplementary file 1 [file genes-14-01379-s001.zip › Figure S3.pdf]

```
DD37E-TRT-Dr   : MGQ----------------KKDLTGSEKSKIVRYLAEG-----------CSTLKIAKLLKRD-------HRTIKRFIQN--SQQGRKKRVEKPRR :  59
DD37E-TRT-Mz   : MGQ----------------KKDLTGSEKSKIVRYLAEG-----------CSSLKIAKLLKRD-------HRTIKRFIQN--SQQGRKKRVEKPRR :  59
DD37E-TRT-Hb   : MGQ----------------KKDLTGSEKSKIVRYLAEG-----------CSSLKIAKLLKRD-------HRTIKRFIQN--SQQGRKKRVEKPRR :  59
DD37E-L18-Acdi : MSK------VNKCGRTYNPGEAIGESLRASIIDRMLLDGGDPATGFFG-GRFKNIGDCFG---VSAPFVSKLWKTFCLQ--GDHMPQKRSSGNPS :  83
DD37E-L18-Exdi : MI-------VTKKGRTFHRGKSLGFDVRTAIVDHMISKGGDIVTXHFP-GSLREVAEHFK---VSKTTVKKIWRQCSET--ADIDVQWKGGNNPP :  82
DD37E-L18-Piim : MKKSH----VNNKGRRYDWGKPLGEDLRSLIVQYLLEKGANSESRFIPRGEMAKAGERFN---VSNNTIKNIWTLYCDS--GDVKHRQGARGRPK :  86
DD37E-MS-Mali  : M------------------QSLNEEQRRRLVVQYKTEHPG---------QSNHAIAKYFAEVGVTRSTVYAILNRYEEGGEESVLRSAGSGCHRT :  68
DD37E-MS-Plmu  : M------------------ARQKSTQLRTAVVNLYNSTLG--------RNRFYQVYLTFKGV-ASKSTIHRIIARSKEH--NNVVAKPRPGRTVK :  66
DD37E-MS-Drin  : M---------------------EKGQRDKIVHNFLENPD----------WSLSKIGKTLK---IAKSTVCDVIKRFKET--HTTNRAKHQTLKRG :  59
DD37E-MS-Opno  : M------------------SSSKELEVRERFGRFMELNPE---------MSATKVVKHFKLEGVPERTGYRIVARIKSG--QGVARNPGSGQNMA :  66
DD37E-MS-Adho  : MMNKKYLDSVSLHSVVFT-MASSEYQQREIILLNHTQKPN---------LSYRALAKLLK---YPQSTVCSVLKKFRER--LTLDRKSGSGGKQG :  80
                              Helix1                         Helix2          Helix3
DD37E-TRT-Dr   : KITAHELRKVKRAAAKMPLATSLAIFQSCNI-----TGVPKSTRCAILRDM----AKVRKAERRPPLNKTHKLKRQDWAKKYLKTDFS------K : 139
DD37E-TRT-Mz   : KITAHELRKVKRAAAKMPLATSLAIFQSCNI-----TGVPKSTRCAILRDM----AKVRKAERRPPLNKTHKLKRQDWAKKYLKTGFS------K : 139
DD37E-TRT-Hb   : KITAHELRKVKRAAAKMPLATSLAIFQSCNI-----TGVPKSTRCAILRDM----AKVRKAERRPPLNKTHKLKRQDWAKKYLKTGFS------K : 139
DD37E-L18-Acdi : HLKPEDVQLVEFLKKEKPSATLASIKETVENYCNLNGGTSLSAIGNTVRNRLPDGPFTRKKLTRPK-AEKFTPQNLAYCGQFLNFISALPPE--K : 175
DD37E-L18-Exdi : HLQSHHLDFIEGLKTIKPSMPYSKIHEAVNMHCPIPAGTSRSSLGKAVQNRLQGGRWTWKRMSRAN-GDKFLPANTNYCQDFLNYMNTIDPY--R : 174
DD37E-L18-Piim : LLDEEDFNYIAAIKKEKPFETLENIKEKLLQ--NANKEVTTMTISNALRKDLIGGQWTRKVLTKTA-TERFTPANEQYTEAYIAEIQSKPPL--R : 176
DD37E-MS-Mali  : ELKVTNAEVKNIVKDAQRGLSQREIARKFDI--------SQAYVGKILQEN---GLKAYKKEKVPAVTDQQRERQQVRVGRLYRTILDGRGGDLD : 152
DD37E-MS-Plmu  : KADAKNLEKLKKIVDGKDHVSDRFAAQRMGW--------SRSYIQKVRVQTL--GIHKYRKRSAPFYTEEEEHEAKKRCRKMVTEVFTPGTN-DL : 150
DD37E-MS-Drin  : CRNRYLRAKVIRSLRANPELSDGDRAKRLKT--------SASTIRRIRLSE---GIKAYVCVKNPNRSDKQNLNAKTRARLLYDQVLTKFQG--C : 141
DD37E-MS-Opno  : -IDQKIKDKIIEFAVNEVGLSYRQIGRKFNV--------SYHSVESILTEA---GVQRVKRKKCPKSTEKQIEAQKKCLQKLRRTLFKASNG-VD : 148
DD37E-MS-Adho  : VKDKKKEARVLQLFTENPKLSSRDVAKKVKM--------SQSYVQKVKSRG---GLKSYKAQAAPNRDAKQNLSAKRRARKLYNDLLTKFE---C : 161
                     Helix1            Helix2                    Helix3
DD37E-TRT-Dr   : VLWTDEMRVSLDGPDGWARGWIGKGQR--APVRLR---RQQGGGGVLVWAGIIKDELVGPFRVEDGVKLNSQSYCQFLEDTFFKQWYRKKSASF- : 228
DD37E-TRT-Mz   : VLWTDEMRVSLDGPDGWARGWIGKGQR--APVRLR---RQQGGGGVLVWAGIIKDELVGPFRVEDGVKLNSQSYCQFLEDTFFKQWYRKKSASF- : 228
DD37E-TRT-Hb   : VLWTDEMRVSLDGPDGWARGWIGKGQR--APVRLR---RQQGGGGVLVWAGIIKDELVGPFRVEDGVKLNSQSYCQFLEDTFFKQWYRKKSASF- : 228
DD37E-L18-Acdi : IKFFDEAGVN-SGT--------GNPVYGSSLKGTKSIEIAAIPRGVNITLNLL-------------VGLEGILYA----NTLDGASNSFTFLNFF : 244
DD37E-L18-Exdi : IKYFDEAGFCLPDV--------GKPTYGHSQINTKCVEIGRYHNAPNVTLNLL-------------IGLXGVVYA----NTEDGPSDTLKFLNFW : 244
DD37E-L18-Piim : LKFMDEAGFALSEA--------VNRTRGHAPKGLRAIEAQKRMKTTNLTLNLM-------------IGLDGTVFS----TFVDGPSNRDEFLKFI : 246
DD37E-MS-Mali  : LVIDDESYFHMSSQMIPGNSFYYATARGDAPVEKRVSPQKKFGDKILVWLAISRHGASRVYMCHS-KTMNKELYA--------NECIRKRLVPFL : 238
DD37E-MS-Plmu  : IVVDDEHYVYLDQSNSVSNMYYNSTSRNVAPKEIKTRPQRKFCPKLMVWLAISVKGRSEPYIAPHKQNIDRKVYL--------KECLEKRLIPFL : 237
DD37E-MS-Drin  : IIMDDETYIKMNFKQLAGRKFYYAYKRGCVASKFKNIPMDKFGRKALIWQAICSCGLKSRAFVTT-SSMTSEVYV--------KECMQKRLLPFI : 227
DD37E-MS-Opno  : IVMDDESYFTIDGSDTNYNDFYYGHPSLEAPESVKYRPIKKFPEKVMVWMAMSPKGFSKPYIVKSGNAVTADVYV--------RESL-TRLRDFL : 234
DD37E-MS-Adho  : VVLDDETYIKADFKQIPGQEFYTSKSRRDAPEDCKVKKRSKFPKKFLLWQAICSCGKRSRSFITT-GTVNGEIYK--------KECLQKRLLPFL : 247
                     D
DD37E-TRT-Dr   : ---------------KKNMIFMQDNAPSHASKY---STAWLARKGIKEEKLMTWPPCSPDLNPIENLWSIIKCEIYKEGKQYTSLNSVWEAVVAA : 305
DD37E-TRT-Mz   : ---------------KKNMIFMQDNAPSHASKY---STAWLARKGIKEEKLMTWPPCSPDLNPIENLWSIIKCEIYKEGKQYTSLNSVWEAVVAA : 305
DD37E-TRT-Hb   : ---------------KKNMIFMQDNAPSHASKY---STAWLARKGIKEEKLMTWPPCSPDLNPIENLWSIIKCEIYKEGKQYTSLNSVWEAVVAA : 305
DD37E-L18-Acdi : GEAGQATSALGNPAIEQGDFIIMDNCAIHRFEAGTALQTWLMDMGANVI---YTPSLSPEFNAVELVFNKLKILLKREEYVPLLKENVHVAIYES : 336
DD37E-L18-Exdi : GEASNSRIPSGFPALQYGDIVVLDNCATHHNNGGFILAEFLDQFGIDVV---YLPVYSPELNPCELAFNKFKKLAQRSDIKAVFNRNVHDGVYEC : 336
DD37E-L18-Piim : DEAATSFSDDGSPVLQPGDVLVVDNATIHRFEAERILRIFFNNIGVEYI---FLPKYSPDMNPVEFSFNYIRTMLKSEIFSALANDNLQYAILKV : 338
DD37E-MS-Mali  : NEQHS----------DGNYLFWPDLASCHYAKD---VIEVFQQLDINFVSKEQNPPCVPHLRPIEHFWGILKQQVYHHSWQAQTLQQLKNRIKKC : 320
DD37E-MS-Plmu  : KTAYP----------KGNAIFWPDKARAHNART---VQFFLREQKIKCVEYNLNPTKFPQARPIETYWAKLDRRLSLYREPTKDLQILKRRIRET : 319
DD37E-MS-Drin  : HMHRS------------PVKFWPDLATCHYSNT---TKLWYEANQVDVLPKWLNPPNCPELRPIERYWAIVKGKLKRSGSIVENEKSMLIAFNKY : 307
DD37E-MS-Opno  : DEYHS----------DENYIFWPDLASSHYART---TQEIYANVGIKVVPKELNPPNVPQLRPIETFWAYLGRKIYANGWTAKNWKQLKRKIEFI : 316
DD37E-MS-Adho  : RNHSS------------QSLFWPDLASCHYSKA---VMEWYDQNSVAVVPKQCNPPNCPELRPIEEYWSIMKGILKKKGGHPKSAAELQKMWTWA : 327
                                        D                                        E
DD37E-TRT-Dr   : ARNVDGEQIRTLTESMDGRLLSVLAKKGG--------YIGH : 338
DD37E-TRT-Mz   : ARNVDGEQIKTLTESMDGRLLSVLAKKGG--------YIGR : 338
DD37E-TRT-Hb   : ARNVDGEQIKTLTESMDGRLLSVLAKKGG--------YIGR : 338
DD37E-L18-Acdi : LDQIDTNDMYGFYSLIL------------------------ : 353
DD37E-L18-Exdi : LQHLTSLDCAGFYREVG--------------------YLSM : 357
DD37E-L18-Piim : LDTISQDDIAGFFSKVG--------------------YIEA : 359
DD37E-MS-Mali  : LREMDWSVVHHMMGQVKSQIRR-ARADGF------------ : 348
DD37E-MS-Plmu  : IKRIPESYLQSVMEGVRTKLRE-IADWGP------------ : 347
DD37E-MS-Drin  : AAKVDRKCVQKLMSNIKKNTRHFIRCKQN------------ : 336
DD37E-MS-Opno  : IRNMPENLCQNLMKNVKTNVRK-AADRGVLSVIKPNQIVSH : 356
DD37E-MS-Adho  : CKKYPDSAVQTLMSRIRRKVRSMAYSKDN------------ : 356
```
