# Supplementary figures and images for: Mosquito (MS), a DD37E Family of Tc1/Mariner, Displaying a Distinct Evolution Profile from DD37E/TRT and DD37E/L18
Source: Genes (Basel). 2023 Jun 29;14(7):1379. doi: 10.3390/genes14071379 (PMC10379824; doi:10.3390/genes14071379)

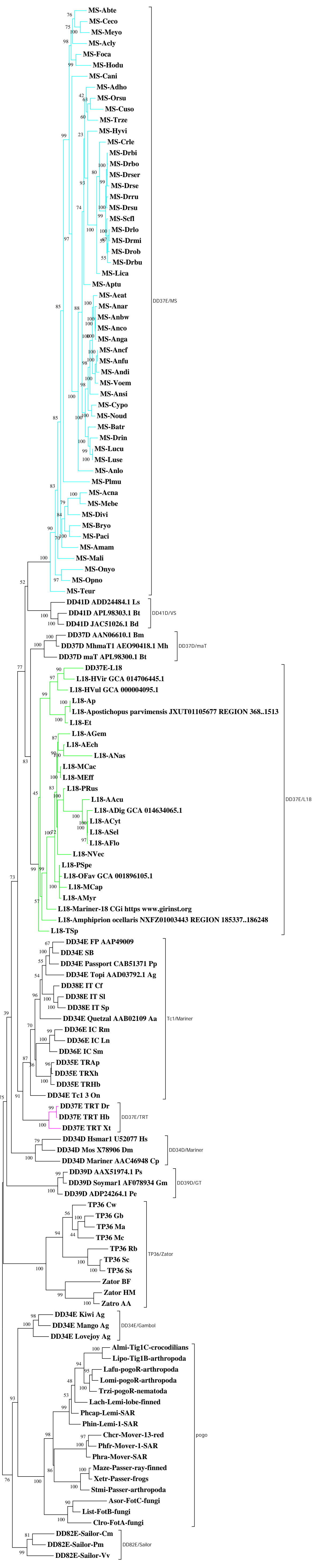

Supplement: Supplementary file 1 [file genes-14-01379-s001.zip › Figure S1.pdf]

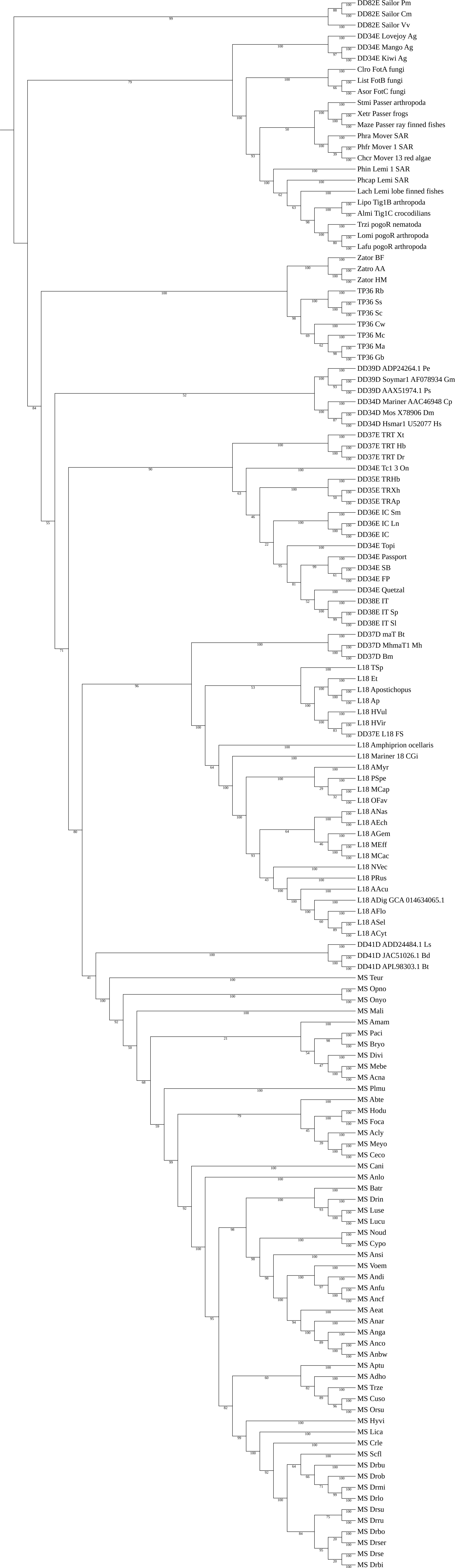

Supplement: Supplementary file 1 [file genes-14-01379-s001.zip › Figure S2.pdf]
